# Red yeast rice extract’s impact on liver health: a pharmacological and metabolomic exploration

**DOI:** 10.3389/fnut.2026.1771594

**Published:** 2026-04-15

**Authors:** Mo Zhao, Yuwei Wang, Yingdong Lu, Zucheng Shang, Xin Zhao, Aimei Lu, Shaoli Wang, Zhen Liu, Hongzheng Li

**Affiliations:** 1 Guang’an Men Hospital, China Academy of Chinese Medical Science, Beijing, China; 2 Institute of Acupuncture and Moxibustion, China Academy of Chinese Medical Sciences, Beijing, China; 3 Health Service Department of the Guard Bureau of the Joint Staff Department, Beijing, China; 4 Dongzhimen Hospital, Beijing University of Chinese Medicine, Beijing, China; 5 Faculty of Medicine, University of British Columbia, Vancouver, BC, Canada

**Keywords:** experimental validation, non-alcoholic fatty liver disease, red yeast rice extract, untargeted metabolomics analysis, Xuezhikang

## Abstract

**Aims:**

This study aimed to investigate the therapeutic mechanism of red yeast rice extract (RYRE) in high-fat diet (HFD)-induced non-alcoholic fatty liver disease (NAFLD) through untargeted metabolomics analysis and experimental validation.

**Methods:**

An untargeted metabolomics analysis based on UHPLC-QTOF/MS was performed to identify differential metabolites in liver tissues. A NAFLD model was established in hamsters by HFD feeding. Forty hamsters were randomly allocated into four groups (*n* = 10 per group): control (CON), model (MOD), red yeast rice extract (Xuezhikang, XZK), and simvastatin (SVT). Serum levels of lipids (TG, CHO, HDL-C, LDL-C), liver function parameters (ALT, AST, ALB, ALP, *γ*-GT, TBiL, DBiL, TBA), and inflammatory cytokines (TNF-*α*, IL-4, IL-1*β*, TGF-β) were measured by biochemical assays and ELISA. Liver tissues were subjected to Oil Red O, hematoxylin–eosin (HE), and Masson staining for histopathological evaluation. Additionally, qPCR and immunohistochemistry were employed to investigate the underlying signaling pathways.

**Results:**

XZK significantly reduced serum levels of DBiL, TBiL, TBA, CHO, TG, and LDL-C, while increasing HDL-C in HFD-fed hamsters. Both XZK and SVT markedly decreased pro-inflammatory cytokines. Untargeted metabolomics identified 135 differential metabolites between the MOD and XZK groups (83 down-regulated, 52 up-regulated), which were primarily involved in carbon metabolism, lipid metabolism, and hormone metabolism. Mechanistically, XZK attenuated the JNK/AP-1/TNF-*α* signaling pathway, as evidenced by reduced mRNA expression of cFos, cJUN, JAK1/2/3, and TNF-α, along with decreased protein levels of p-JNK, cFos, cJUN, and TNF-α. Notably, the p-cJun/total cJun ratio showed a distinct regulatory pattern, suggesting complex modulation of AP-1 subunit phosphorylation.

**Conclusion:**

XZK effectively ameliorates hepatic steatosis, dyslipidemia, and inflammation in HFD-induced NAFLD hamsters. The therapeutic effects are mediated through restoration of metabolic homeostasis and suppression of the JNK/AP-1/TNF-*α* signaling pathway.

## 1 Introduction

Non-alcoholic fatty liver disease (NAFLD), recognized as one of the most prevalent chronic liver disorders, is defined by the excessive accumulation of triglycerides (TG) as lipid droplets within the cytoplasm of hepatocytes, occurring in the absence of significant alcohol consumption (1). NAFLD constitutes a significant risk factor for the progression to cirrhosis and potentially subsequent hepatocellular carcinoma (2, 3). The pathogenesis of NAFLD initiates with the excessive deposition of TG in the liver, which may advance to non-alcoholic steatohepatitis (NASH) (4). The global prevalence of NAFLD has escalated rapidly, attributable to shifts in lifestyle and dietary practices. NAFLD, recognized as one of the most prevalent chronic liver conditions, is characterized by the excessive accumulation of TG as lipids. The development and progression of NAFLD are closely linked to obesity and the consumption of diets rich in sugar and fat. Recent research involving both human and animal models has identified high fructose intake as a significant contributor to hepatic steatosis (5). The excessive intake of fructose leads to increased lipid accumulation in the liver through various mechanisms, with enhanced lipid synthesis playing a crucial role (6).

Traditional Chinese Medicine (TCM) has a longstanding history in the clinical treatment of NAFLD (7–9). Red yeast rice extract (RYRE) has been utilized for centuries in China and other Asian countries as a traditional medicine, serving as a food coloring, additive, and preservative (10). RYRE naturally comprises several constituents that may assist in regulating cholesterol levels, including various monacolins, sterols, isoflavones, and monounsaturated fatty acids, which are believed to contribute to cholesterol reduction (11–13). Xuezhikang (XZK), a patented Chinese medicine comprising an RYRE, is a prevalent herbal component in TCM formulations, such as the Baohe pill, which are employed in the treatment of hyperlipidemia and coronary heart disease (14, 15). Notably, the clinical efficacy of XZK in ameliorating dyslipidemia and reducing cardiovascular risk has been firmly established in large-scale human trials, such as the China Coronary Secondary Prevention Study (16–18). However, its precise mechanisms of action, particularly in the context of NAFLD, remain incompletely understood. This study therefore aims to investigate the therapeutic effects of XZK on NAFLD and explores potential mechanisms through untargeted metabolomics analysis.

## 2 Materials and methods

### 2.1 Materials and reagents

XZK was purchased from Beijing Peking University WBL Biotech CO., LTD (Z10950029, Cat: 20220636, Beijing, China) and dissolved in normal saline. CHO (NO. S03027), TG (NO. S03042), HDL-C (NO. S03025), LDL-C (NO. S03029), ALT (NO. S03030, AST (NO. S03040), ALB (NO. S03043), ALP (NO. S03038), *γ*-GT (NO. S03031), TBA (NO. S03074) kits were purchased from Rayto Life and Analytical Sciences Co., Ltd. (Shenzhen, China). TBiL (NO. C120), DBiL (NO. C119) were purchased from Changchun Huili Biotech Co., Ltd. (Changchun, China). TNF-*α* (cat: EK382-01), TGF-*β* (cat: EK981-01), IL-4 (cat: EK304-01), IL-1β (cat: EK301BHS-01) rat kits were purchased from Multisciences (Lianke) Biotech Co., Ltd. (Hangzhou, China).

### 2.2 Animals and drug administration

Forty SPF-grade male golden hamsters [6-week-old, certificate No. SCXK (Jing) 2021–0011] were provided by Beijing Vital River Laboratory Animal Technology Co., Ltd. (Beijing, China). The hamsters were kept in a controlled environment at 21–25 °C and 40–50% humidity with a 12-h light/dark cycle for 7 days. They had free access to water and a normal diet. The animal facility was provided by the Institutional Animal Care and Use Committee of Liaoweiyuan (Beijing) Pharmaceutical Technology Co., Ltd. (Approval No. SYXK2022-0007). The animal protocol was approved by Xiyuan Hospital, China Academy of Chinese Medical Sciences (Approval No. 2023XLC017-2).

After acclimatization, 10 hamsters were fed a standard diet (CON group), while the remaining 30 hamsters were fed a high-fat diet (HFD) for 8 weeks to induce the NAFLD model. At the end of the 8-week induction period, blood was collected from the orbital sinus to measure serum CHO and TG levels, and abdominal ultrasound was performed to observe hepatic pathological changes. The 30 HFD-fed hamsters were then randomly divided into three groups (*n* = 10 per group): model (MOD, treated with water), XZK (0.205 g/kg/day, Beijing Peking University WBL Biotech Co., Ltd., Z10950029), and simvastatin (SVT, 1.71 mg/kg/day, China Resources Double-Crane Pharmaceutical Co., Ltd., H20065196). Hamsters in the XZK and SVT groups received daily oral gavage at 9:00 a.m. for 4 weeks, while those in the CON and MOD groups received an equal volume of saline solution. During the treatment period, the CON group continued on a standard diet, whereas the MOD, XZK, and SVT groups remained on the HFD. The HFD (D12109C, Xiao Shu You Tai (Beijing) Biotechnology Co., Ltd., SCXK(Jing) 2018–0006) contained 20% protein, 40% carbohydrate, and 40% fat.

### 2.3 Sample of hamsters collection

After 4 weeks of gavage, the hamsters were fasted for 8 h and were anesthetized with 0.1% sodium pentobarbital, and blood was drawn from the aorta abdominalis. Serum was separated by centrifugation at 3500 rpm for 20 min at 4 °C (Sigma 3–18, Germany) and stored at −80 °C freezer for subsequent assays. The hamsters underwent laparotomies along the abdomen’s midline to the sternum’s angle, the liver samples were carefully excised and weighed, immediately snap frozen using liquid nitrogen, and then stored at −80 °C for metabolomic analysis. A viscera index was calculated using the formula: organ weight/body weight (mg/g).

### 2.4 Biochemical assay of blood

CHO (Rayto, NO. S03027), TG (Rayto, NO. S03042), HDL-C (Rayto, NO. S03025), and LDL-C (Rayto, NO. S03029) levels in the blood serum were measured to evaluate the expression of lipid substances. ALT (Rayto, NO. S03030, AST (Rayto, NO. S03040), TBiL (Changchun Huili, NO. C120), DBiL (Changchun Huili, NO. C119), ALB (Rayto, NO. S03043), ALP (Rayto, NO. S03038), *γ*-GT (Rayto, NO. S03031), TBA (Rayto, NO. S03074) levels in the blood serum were measured to evaluate liver function. TNF-*α* (Multisciences, EK382-01), TGF-*β*(Multisciences, EK981-01), IL-4 (Multisciences, EK304-01), IL-1β (Multisciences, EK301BHS-01) levels in the blood serums were measured to evaluate the anti-inflammatory function of the hamsters.

### 2.5 Histological examination of liver

After the frozen sections of liver were fixed, oil red O staining, background differentiation, hematoxylin staining and sealing were performed in turn. Lipid deposition in liver was observed by oil red O staining. HE staining examined the degree of liver tissue damage. CaseViewer 2.2 scanning software was applied to select the liver area for 200x imaging. During imaging, try to fill the entire field of vision with tissues to ensure that the background light of each photograph is consistent. After imaging, use Image Pro Plus 6.0 analysis software to measure the pixel area of oil red grease drops in each picture and the corresponding pipe wall pixel area and calculate the proportion of oil red grease drops in a unified unit pixel.

### 2.6 Hybrid Quadrupole-Orbitrap™ Mass Spectrometer for metabolomics analysis in liver tissues

Liver sample weighing 80 mg was transferred to 2 mL grinding tube containing 50 mg Zirconia beads. Liver homogenate were obtained by addition of 200 μL of ultra pure water to grinding tube and homogenized for 30 s in low temperature grinder at maximum speed. The homogenized samples added of 400 μL methanol: acetonitrile (volumetric ratio of 1:1) to vortex 30 s, then extract for 30 min by ultrasonic extractor at low temperature. The homogenized samples were then centrifuged for 20 min at 13500 g, after which the supernatant was transferred to EP tubes and blow dry with nitrogen blowing instrument. Take 20 mg of each group of samples and mix them into QC liver samples, and use the same preparation method to prepare QC samples. Finally, the residue was reconstituted with 200 μL methanol: acetonitrile (1:1), centrifuged for 20 min at 13500 g, and an aliquot of 3 μL was injected for Hybrid Quadrupole-Orbitrap™ Mass Spectrometer analysis.

Samples were analyzed using an Thermo Scientific Ultimate 3,000 system coupled to an Thermo Scientific QExactive plus (Hybrid Quadrupole-Orbitrap™ Mass Spectrometer) with Waters BEH C18 (1.7 μm, 100 mm) refer to previous methods (19–21). Eluent A consisted of 0.1% formic acid (v/v) in water and eluent B consisted of 0.1% formic acid (v/v) in 100% acetonitrile. The analytical gradient was: 0 min, 1% A; 2 min, 35% A; 7 min, 75%A; 10 min, 99% A; 11 min, 99% A; 16 min, 1% A. Flow rate was 0.4 mL/min with an injection volume of 3 μL. Samples were held at 4 °C in the auto-sampler, and the column was operated at 45 °C. The MS operated in positive ionization mode and negative ionization mode with capillary voltage set to 3.5 KV and 2.8 KV. Capillary temperature and aux gas heater temperature in positive and negative ion mode were both set to 320 °C. Scan range was150–1,200 m/z.

### 2.7 Real-time PCR analysis

The RT-PCR was performed to quantify the expression of cFos, cJUN, JAK1/2/3, TNF-*α*. The process was the same as in our previous study. The results are presented as relative quantity to GAPDH, which served as an internal control. The RNA primer were as followed: cFos-F (AACTGACTGATACTCTCCAA), cFos-R (AAGCCACAGACATCTCTT), cJUN-F (CCTACGGCTACAGTAACC), cJUN-R (GAGATGCGGCTTCAGATT), JAK1/2/3-F (GGAATCAAGCACCTTCAC), JAK1/2/3-R (GTAGCGAGTCACCACATA), TNF-*α*-F (TGAGCCATCGTGCCAATG), TNF-*α*-R (AGCCCGTCTGCTGGTATCAC), GAPDH-F (GTATTGGACGCCTGGTTA), GAPDH-R (CCTGGAAGATGGTGATGG).

### 2.8 Immumohistochemical staining

For immunofluorescence staining, the sections were steamed in citrate buffer for antigen retrieval, and blocked using PBS containing 1% (w/v) bovine serum albumin (BSA). The slides were incubated with the corresponding antibody (cFos, 1:100, Affinity, Cat. AF5354; cJUN, 1:100, Affinity, Cat. AF6090; JNK1/2/3, 1:100, Affinity, Cat. AF6318; TNF-*α*, 1:50, Affinity, Cat. AF7014) at a dilution of 1/100 overnight at 4 °C. Then, the sections were incubated with anti-rabbit antibody labeled with secondary antibodies (Goat anti-rabbit IgG H&L(HRP), 1:20000, abcam, Cat. Ab205718) for 1 h. The nuclei were visualized by Fluoroshield mounting medium with DAPI (Beyotime Biotechnology, Shanghai, China) staining.

Immunohistochemistry analysis were performed using Image ProPlus 6.0 software (Media Cybernetics Inc., Silver Spring, MD, United States) for semiquantitative analysis.

### 2.9 Statistical analysis

The raw data from the experiment were processed using Progenesis QI v2.0 (Non-linear Dynamics, Newcastle, United Kingdom) to facilitate the visualization, processing, and interpretation of multidimensional liquid chromatography-mass spectrometry (LC–MS) data. The LC–MS data were imported into Progenesis QI for peak picking and alignment, which enabled the generation of principal component analysis (PCA) and orthogonal partial least squares-discriminant analysis (OPLS-DA). These analyses were subsequently validated through analysis of variance (ANOVA). Metabolite peaks were identified through tandem mass spectrometry (MS/MS) analysis, supplemented by the Mass Fragment TM application manager (Waters Corporation, Milford, United States). Bioinformatics analyses were performed using established biochemical databases, including the Human Metabolome Database (HMDB)^1^ and the Kyoto Encyclopedia of Genes and Genomes (KEGG)^2^. Additional multivariate statistical analyses were conducted using SIMCA software (Version 14.1, MKS Data Analytics Solutions, Umea, Sweden). OPLS-DA was employed to visualize group differences and to identify the variables contributing to group separation.

The precise significance values are provided in all figures, with data presented as means ± standard deviation in the figure legends. Comparisons between the two conditions were conducted using unpaired Student’s t-tests. Graphs and statistical analyses were generated using GraphPad PRISM version 8.0. Differences were deemed statistically significant at *p* < 0.05 and *p* < 0.01.

## 3 Results

### 3.1 XZK reduced lipid levels in serum of NAFLD hamsters

**Figure 1:**
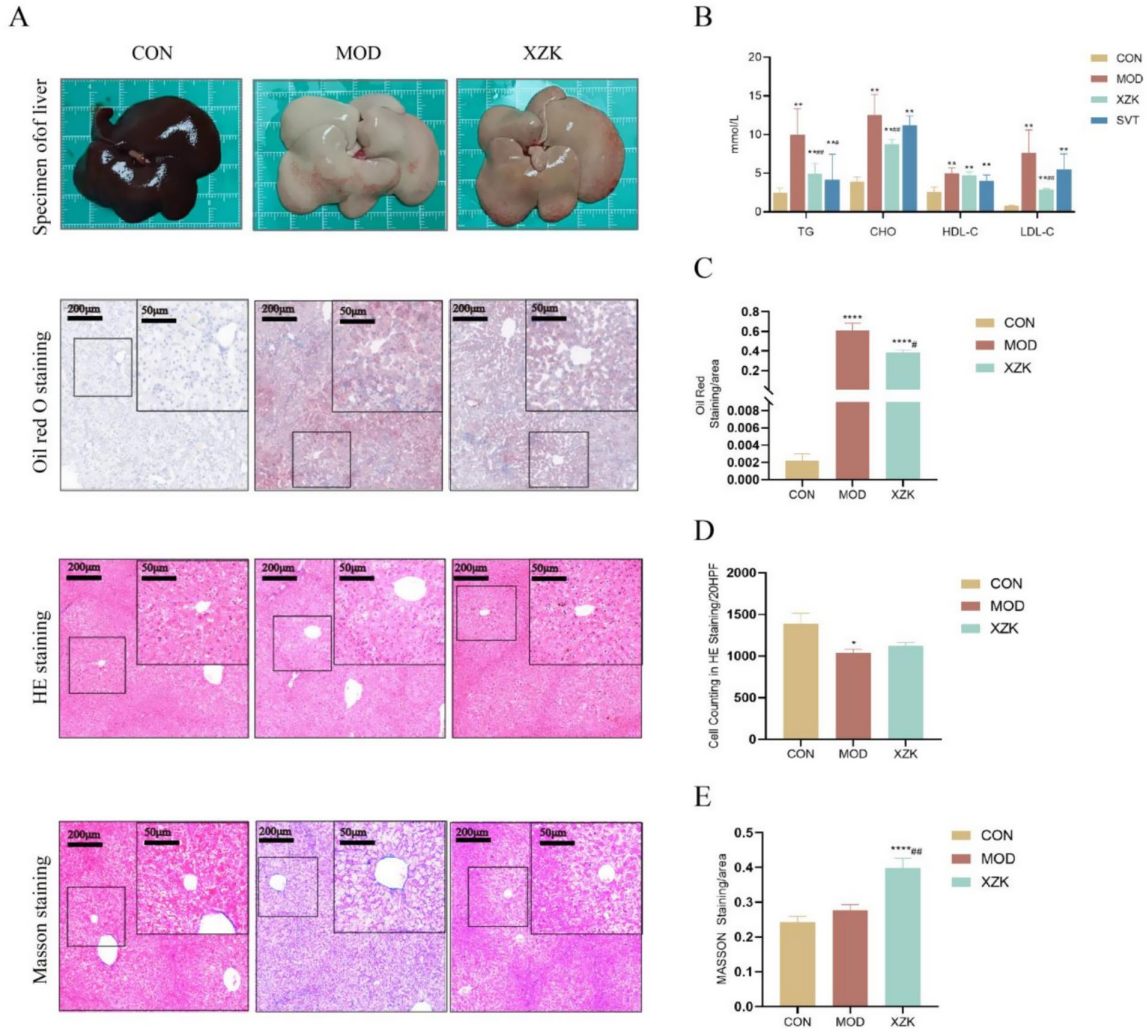
The degree of liver damage and lipid level. **(A,C**–**E)** Liver tissues of hamsters. Oil red O, HE, and Masson’s staining of livers (original magnification 40x, scale bar = 50 μm, *n* = 10 per group). Phenotype, ballooning, lipid accumulation, and Masson’s trichrome staining are shown in **(C–E)**. **(B)** Lipid index include TG, CHO, HDL-C, and LDL-C levels in the serum. *p < 0.05, **p < 0.01, ***p < 0.001, ****p < 0.0001 via CON group; #*p* < 0.05, ##*p* < 0.01 via the MOD group.

Following a 4-week intervention with XZK, liver histopathology and serum biochemical parameters were evaluated. Histochemical staining (Oil Red O, H&E, and Masson) revealed that XZK treatment significantly alleviated hepatic lipid accumulation induced by the high-fat diet (HFD; Figure 1A). Quantitative analysis of the staining further confirmed that HFD caused substantial pathological alterations, which were partially but significantly reversed by XZK (*p* < 0.05, *p* < 0.01; Figures 1C,E). In line with the histological improvements, XZK intervention led to a marked reduction in key serum lipid parameters. Specifically, levels of triglycerides (TG), total cholesterol (CHO), and low-density lipoprotein cholesterol (LDL-C) were all significantly decreased (*p* < 0.01 for all), while high-density lipoprotein cholesterol (HDL-C) remained largely unchanged (Figure 1B). Notably, the HFD-fed hamsters in our model primarily developed hepatic steatosis (lipid accumulation), without exhibiting widespread advanced pathological features such as severe ballooning degeneration or bridging fibrosis (Figure 1D). This is likely attributable to the relatively short (7-week) induction period, which was sufficient to induce metabolic lipid accumulation but not extensive hepatocyte injury or fibrogenesis. In summary, XZK treatment effectively reduced hepatic steatosis, improved serum lipid profiles (lowering TG, CHO, and LDL-C), and mitigated HFD-induced pathological damage in the liver.

### 3.2 XZK alleviated mild liver injury and inflammation in NAFLD hamsters

**Figure 2:**
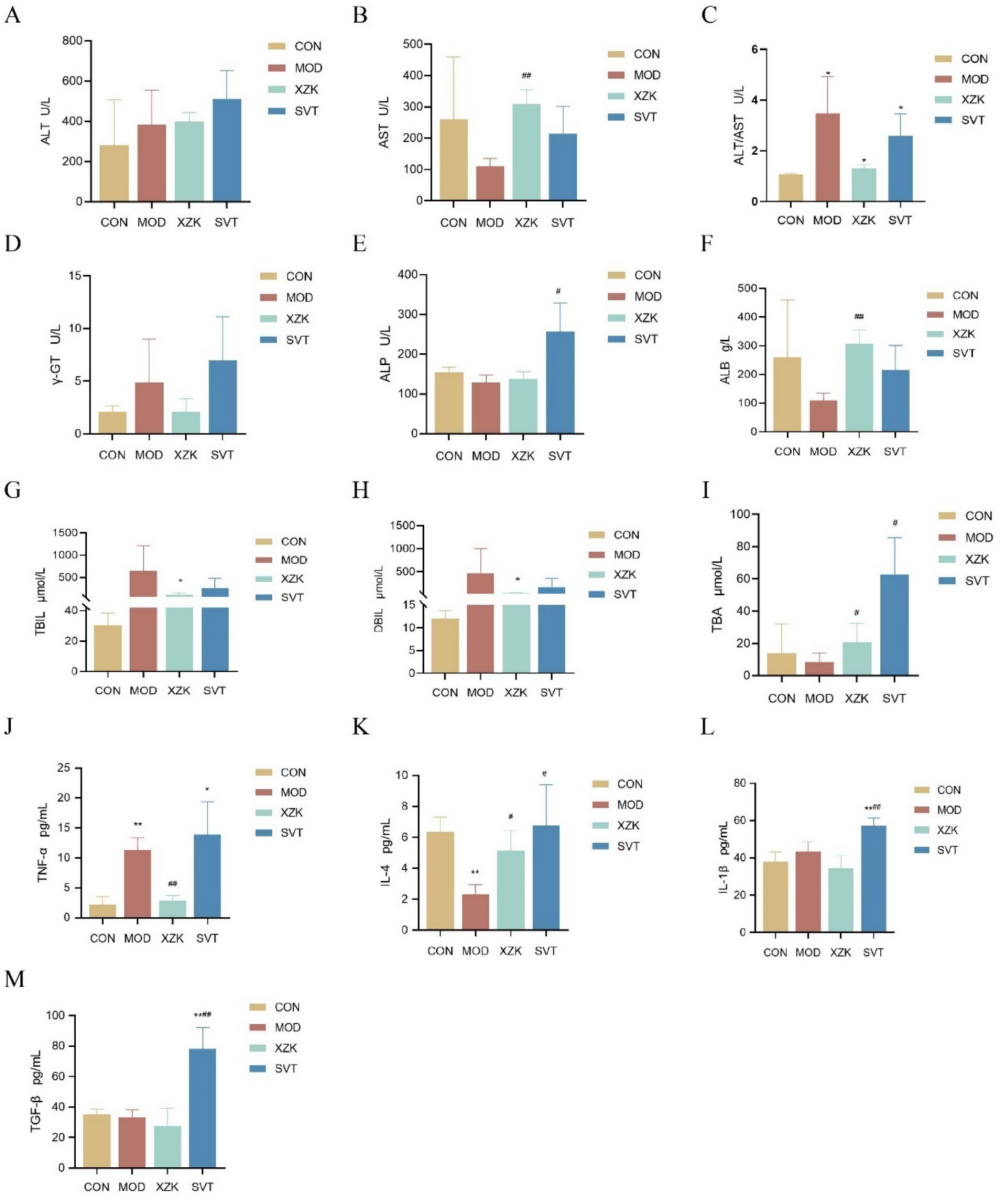
Expression of liver functional and inflammatory factors in serum (*n* = 6). **(A–I)** Expression of ALT, AST, ALT/AST, ƴ-GT, ALP, ALB, TBiL, DBiL, TBA in serum. **(J**–**M)** Expression of inflammation indexes TNF-*α*, IL-4, IL-1*β*, and TGF-*β* in serum. *p < 0.05, **p < 0.01 via CON group; #*p* < 0.05, ##*p* < 0.01 via MOD group.

To evaluate the hepatoprotective and anti-inflammatory effects of the interventions, we measured a panel of serum biomarkers. Liver function parameters, including ALT, AST, the ALT/AST ratio, ƴ-GT, ALP, ALB, TBiL, DBiL, and TBA, were assessed (Figures 2A–I). XZK treatment significantly modulated several key indices, notably the ALT/AST ratio, ALB, TBiL, DBiL, and TBA levels (*p* < 0.05 for all). The elevated bilirubin levels (TBiL, DBiL) in the HFD group, indicative of liver dysfunction and impaired biliary excretion commonly seen in NAFLD, were effectively reduced by XZK. Concurrently, we measured serum levels of pro- and anti-inflammatory cytokines (TNF-*α*, IL-4, IL-1*β*, TGF-*β*; Figures 2J–M). Both XZK and simvastatin (SVT) significantly suppressed pro-inflammatory factors. Notably, the reduction in TNF-*α* and IL-4 emerged as a primary characteristic of XZK’s action in alleviating HFD-induced liver injury.

Collectively, these findings demonstrate that XZK confers potent hepatoprotective effects. The amelioration of liver enzyme profiles and bilirubin levels, coupled with the suppression of key inflammatory mediators such as TNF-*α*, indicates a comprehensive improvement in liver function and a reduction in hepatocellular damage. This dual action on metabolic and inflammatory pathways underscores the therapeutic potential of XZK in NAFLD.

### 3.3 Metabolites with significant regulation with the treatment of XZK

**Figure 3:**
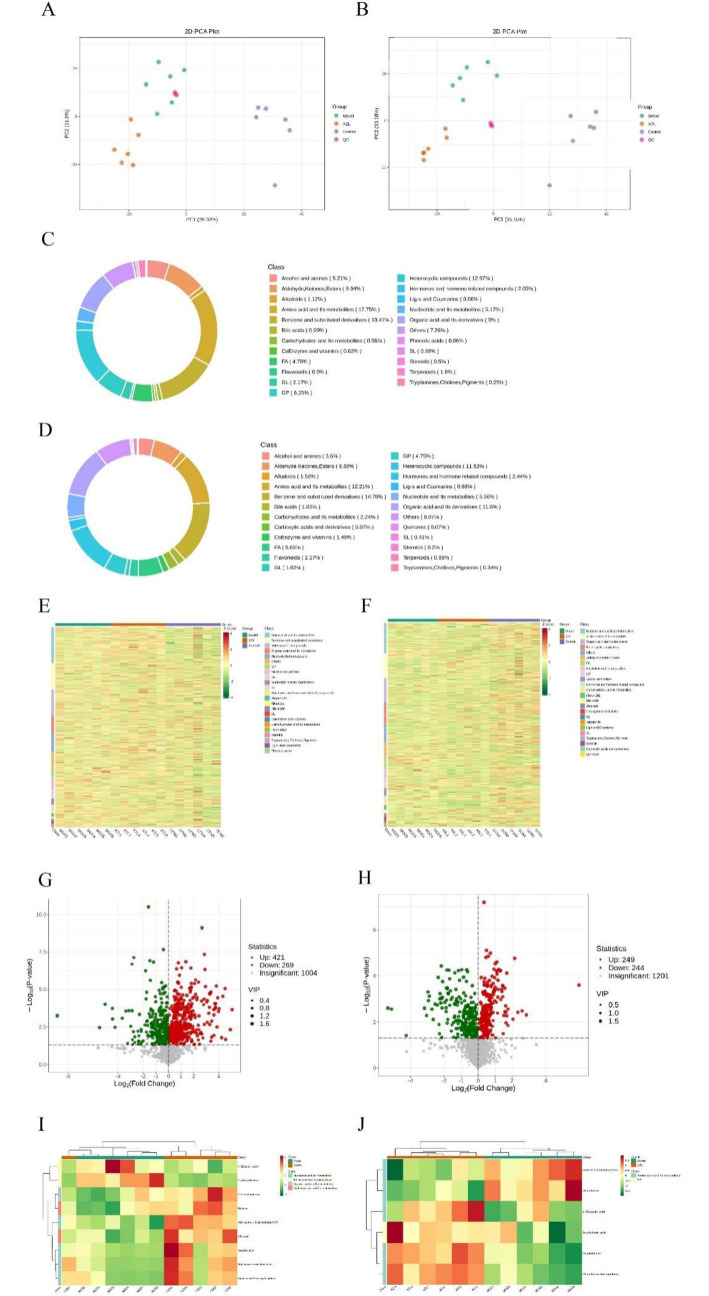
Multivariate statistical analysis of the LC-MS spectra of hamster cecal contents taken from the three groups (*n* = 6). **(A**,**B)** PCA score map of the three groups. **(C)** Bar chart of different metabolites between CON and MOD. **(D)** Bar chart of different metabolites between MOD and XZK. **(E)** Correlation heatmap between CON and MOD. **(F)** Correlation heatmap between MOD and XZK. **(G)** Differential volcanic map of CON vs. MOD group. **(H)** Differential volcanic map of XZK vs. MOD group. **(I)** Differential heatmap of CON vs. MOD group. **(J)** Differential heatmap of XZK vs. MOD group. PC1 represents the first principal component and PC2 represents the second principal component. Percentage represents the interpretation rate of the principal component to the dataset and the ellipse represents a 95% confidence interval. Each dot in the figure represents a sample, and samples in the same group are indicated in the same color.

To achieve a comprehensive understanding of the metabolic alterations associated with the NAFLD model, we conducted untargeted metabolite profiling of liver tissues. This profiling provided significant insights into the metabolic changes induced by a high-fat diet (HFD) and the subsequent therapeutic effects of XZK. The metabolic composition of hamster serum was analyzed using both positive and negative ion modes via liquid chromatography-mass spectrometry (LC/MS), with representative total ion chromatograms for the quality control (QC) samples depicted in Supplementary Figure 1. Principal component analysis (PCA), an unsupervised multivariate statistical method, was employed to assess the overall differences among samples within each group and to evaluate the degree of variability among the samples collectively. The PCA results demonstrated notable separation of gastrointestinal contents among the CON, MOD, and XZK groups in both negative and positive ion modes (Figures 3A–D). This separation indicates that the samples in the MOD group exhibited deviations from normal metabolic levels, reflecting metabolic disorders, while the XZK group showed a gradual recovery of abnormal metabolic processes (Figures 3E,F).

To elucidate the similarities and differences between the two groups and enhance the model’s effectiveness and analytical capabilities, Orthogonal Partial Least Squares Discriminant Analyses (OPLS-DA) were conducted to identify potential biomarkers, as depicted in Supplementary Figure 2. To ensure the reliability of the results, the OPLS-DA model underwent 200 permutation tests, with the intercept of the Q2 regression line being less than zero, indicating that the model was not overfitting (Supplementary Figure 2). Subsequently, heatmap was used to display the trend patterns of differential expression of metabolites between the groups (Figures 3I,J), while volcano plots were utilized to illustrate the upregulation or downregulation of metabolites between the different groups, as shown in Figures 3G,H. Metabolites in liver samples were identified using mass spectrometry data, KEGG, and HMDB 4.0. Potential biomarkers were identified based on a Variable Importance in Projection (VIP) value greater than 1.0 and statistical significance (*p* < 0.05). A total of 275 metabolites exhibited significant differences between the CON and MOD, as well as between the XZK and MOD groups. Furthermore, 506 metabolites showed significant differences between the CON and MOD groups and the XZK and CON groups, while 331 metabolites were significantly different between the XZK and MOD groups and the XZK and CON groups. In comparison to the CON vs. MOD group, 135 metabolites were significantly altered in the MOD vs. XZK group, with 83 metabolites being down-regulated and 52 metabolites being up-regulated. The alterations in these potential biomarkers are detailed in Table 1. The metabolites detected in each group and the comparison among groups were showed in Supplementary Table 1.

**Table 1:** Different metabolites between three groups.

Class I	Class II	Mode	Compounds	Formula	Q1 (Da)	Molecular weight (Da)	RT (min)	Adduct	Mass error	Level	score	Con vs. Mod	Mod vs. XZK	*p*
Alcohol and amines	Amines	positive	Alpha-Linolenoyl ethanolamide	C20H35NO2	385.2743	321.2668	8.1836	M + CH3CN + Na	21.7328	2	0.5135	up	down	0.004
		positive	Agomelatine	C15H17NO2	244.1365	243.1259	5.1166	M + H	8.4204	2	0.921	up	down	0.048
	Alcohols	positive	2-Aminooctadec-4-ene-1,3-diol	C18H37NO2	322.2722	299.2824	6.8576	M + Na	1.1750	3	0.9998	up	down	0.025
		negative	9xi-Episterol	C28H46O	433.3266	398.3549	9.1582	M + Cl	5.3770	3	0.8734	up	down	0.008
Aldehyde, Ketones, Esters	Aldehydes	positive	5-Hydroxymethyl-2-furancarboxaldehyde	C6H6O3	275.053	126.0317	0.8041	2 M + Na	2.3027	2	0.7317	up	down	0.012
		negative	2-trans,6-trans-Farnesal	C15H24O	255.16	220.1827	6.7724	M + Cl	19.7000	3	0.5352	up	down	0.000
	Esters	positive	(3S,5S)-3,5-Diaminohexanoate	C6H14N2O2	293.2093	146.1055	8.8812	2 M + H	22.6500	3	0.6123	up	down	0.002
		positive	HoPhe-Tyr-OH	C24H22N2O7	451.1502	450.1427	5.2079	M + H	3.9537	2	0.8654	up	down	0.009
		negative	Leukotriene C4 methyl ester	C31H49N3O9S	638.32	639.319	8.9496	M-H	14.4866	2	0.694	down	up	0.027
		negative	[(2R,3R,4S,5S,6R)-2-[(1R,2Z,3S,4R,5S)-2-(cyanomethylidene)-3-hydroxy-4,5-dimethoxycyclohexyl]oxy-4,5-dihydroxy-6-(hydroxymethyl)oxan-3-yl] (Z)-3-(4-hydroxy-3-methoxyphenyl)prop-2-enoate	C26H33NO12	550.1883	551.2003	7.8653	M-H	8.4164	2	0.8517	down	up	0.008
	Ketones	negative	2-Methylcyclododecanone	C13H24O	241.1807	196.1827	6.9977	M + HCOO	2.0861	3	0.719	up	down	0.001
		negative	Licofelone	C23H22ClNO2	419.1504	379.1339	11.1519	M-H + CH3CN	3.8397	2	0.6956	down	up	0.015
		negative	(3S,7R)-11-methoxy-3,7-dimethyl-6,8,16,20-tetraoxapentacyclo[10.8.0.02,9.03,7.013,18]icosa-1,4,9,11,13(18)-pentaene-17,19-dione	C19H16O7	337.0705	356.0896	5.1706	M-H2O-H	3.0000	3	0.9429	down	up	0.002
Alkaloids	Alkaloids	positive	Ergosine	C30H37N5O5	548.2752	547.2795	8.1836	M + H	20.8024	2	0.8021	up	down	0.002
		positive	(9R)-5-bromo-N-[(2S,4R,7S)-2-hydroxy-7-(2-methylpropyl)-5,8-dioxo-4-propan-2-yl-3-oxa-6,9-diazatricyclo[7.3.0.02,6]dodecan-4-yl]-7-methyl-6,6a,8,9-tetrahydro-4H-indolo[4,3-fg]quinoline-9-carboxamide	C32H40BrN5O5	654.2445	655.2198	5.2079	M + H	24.7340	2	0.8176	up	down	0.001
		negative	Ochrolifuanine A	C29H34N4	437.2722	438.2783	9.6617	M-H	5.0388	3	0.7241	down	up	0.031
Amino acid and Its metabolites	Amino acids	positive	L-Histidine	C6H9N3O2	156.0765	155.0695	0.6209	M + H	0.7460	1	0.9506	up	down	0.035
	Amino acid derivatives	negative	Adouetine X	C28H44N4O4	499.3331	500.3363	8.2786	M-H	7.5581	3	0.6799	down	up	0.000
	Small Peptide	positive	Leu-Gly-Asp-Val-Ile	C23H41N5O8	516.3061	515.2955	7.9857	M + H	6.3810	2	0.8979	down	up	0.002
		positive	Leu-Arg-Asn-Arg	C22H43N11O6	558.3534	557.3398	9.551	M + H	10.7443	2	0.8922	down	up	0.009
		positive	Tyr-Phe-Val-Arg	C29H41N7O6	584.3124	583.3118	8.5925	M + H	13.1420	2	0.8906	up	down	0.024
		positive	Gln-Tyr-Val	C19H28N4O6	409.206	408.2009	5.7283	M + H	4.6377	2	0.8892	up	down	0.043
		positive	Ile-Asp-Phe-Arg	C25H39N7O7	550.2911	549.2911	8.7135	M + H	13.2748	2	0.864	up	down	0.011
		positive	Leu-Nap-OH	C24H24N2O6	437.1712	436.1634	5.2079	M + H	3.4094	2	0.8461	up	down	0.001
		positive	Thr-Leu-Lys	C16H32N4O5	361.2358	360.2373	8.0542	M + H	19.7538	2	0.8383	up	down	0.000
		positive	Pro-Leu-Glu-Gly-Lys	C24H42N6O8	543.3254	542.3064	8.3652	M + H	19.5577	2	0.8149	down	up	0.000
		positive	Lys-Gln-Ile-Glu	C22H40N6O8	517.3098	516.2908	7.9918	M + H	21.1417	2	0.8116	down	up	0.002
		positive	Arg-Ala-Glu-Lys	C20H38N8O7	503.2966	502.2863	8.1986	M + H	5.3072	2	0.7502	up	down	0.241
		positive	Leu-Lys-His-Glu	C23H39N7O7	526.2933	525.2911	8.1836	M + H	9.5864	2	0.7418	up	down	0.005
		positive	Thr-Lys-Gln-Lys	C21H41N7O7	504.3089	503.3067	8.547	M + H	9.5399	2	0.7412	up	down	0.005
		positive	Phe-Met-Val	C19H29N3O4S1	459.2066	395.1879	5.7283	M + CH3CN + Na	5.3675	2	0.5916	up	down	0.007
		negative	MET-enkephalin	C27H35N5O7S	572.2307	573.2257	8.5526	M-H	21.4307	2	0.7195	down	up	0.002
		negative	Phe-Asp-Glu-Phe-Leu	C33H43N5O10	668.2953	669.301	5.2308	M-H	4.7261	2	0.8484	up	down	0.044
		negative	Leu-Glu-Phe-Glu	C25H36N4O9	535.2405	536.2482	7.8653	M-H	1.6013	2	0.8292	down	up	0.001
		negative	His-Ala-Arg-Glu	C20H33N9O7	510.237	511.2503	8.568	M-H	7.6385	2	0.7714	down	up	0.003
		negative	Leu-Tyr-Gln-Glu	C25H37N5O9	550.2507	551.2591	8.568	M-H	3.3770	2	0.723	down	up	0.001
		negative	Thr-Phe-Val-Arg	C24H39N7O6	580.3167	521.2962	8.5066	M + CH3COO	9.2760	2	0.6982	up	down	0.019
		negative	Arg-Phe-Tyr-Arg	C30H44N10O6	639.3249	640.3445	8.6596	M-H	16.1894	2	0.6569	down	up	0.045
		negative	Asp-Leu-Phe-Arg	C25H39N7O7	548.2974	549.2911	9.1343	M-H	24.3035	2	0.6183	down	up	0.008
Benzene and substituted derivatives	Benzene and substituted derivatives	positive	Etobenzanid	C16H15Cl2NO3	378.0144	339.0429	5.6979	M + K	17.6159	2	0.654	up	down	0.003
		positive	Deoxynojirimycin Tetrabenzyl Ether	C34H37NO4	524.2778	523.2723	7.8026	M + H	2.9657	2	0.7488	up	down	0.012
		negative	m-Chlorohippuric acid	C9H8ClNO3	425.0351	213.0193	5.1706	2 M-H	4.1663	2	0.6326	down	up	0.002
		negative	Buprenorphine	C29H41NO4	466.2941	467.3036	9.1343	M-H	4.7995	2	0.7823	down	up	0.002
		negative	Integerrine	C35H39N5O4	592.2957	593.3002	8.4152	M-H	3.5035	3	0.6806	down	up	0.000
		negative	4-Hydroxyphenylpyruvic acid	C9H8O4	220.0628	180.0423	2.9045	M-H + CH3CN	4.5500	3	0.8863	up	down	0.031
		negative	Candesartan cilexetil	C33H34N6O6	609.2566	610.254	8.2027	M-H	14.3890	2	0.811	up	down	0.026
		negative	Arachidonoyl p-Nitroaniline	C26H36N2O3	483.2962	424.2726	5.2459	M + CH3COO	19.2819	2	0.8111	up	down	0.046
		negative	Montelukast sulfoxide	C35H36ClNO4S	600.2044	601.2054	8.2482	M-H	9.7353	2	0.8608	up	down	0.019
		negative	Tetracenomycin E	C22H16O8	460.0699	408.0845	1.1299	M + Cl + NH3	22.9645	3	0.5195	up	down	0.002
		negative	Hydroxybuprenorphine	C29H41NO5	464.2781	483.2985	8.3696	M-H2O-H	5.2478	3	0.5109	down	up	0.014
	Phenolics	positive	1,2,4-Benzenetriol	C6H6O3	275.0529	126.0317	1.0463	2 M + Na	2.3463	2	0.7252	up	down	0.024
		positive	N-Cyclopropyl-11-(2-hexyl-5-hydroxyphenoxy)undecanamide	C26H43NO3	502.2935	417.3243	8.1986	M + K + HCOOH	0.3968	2	0.8787	up	down	0.022
Carbohydrates and Its metabolites	Sugars	positive	Lewis A trisaccharide	C20H35NO15	568.171	529.2007	4.1352	M + K	11.8357	2	0.6849	down	up	0.018
	Carbohydrates and Its metabolites	negative	Sedoheptulose	C7H14O7	245.0429	210.074	0.8248	M + Cl	0.3572	1	0.7364	up	down	0.047
FA	Oxidized lipids	positive	7-[2-(3-Hydroxyoctyl)-5-oxopyrrolidin-1-YL]heptanoic acid	C19H35NO4	342.2705	341.2566	10.3357	M + H	14.4056	2	0.7704	up	down	0.028
	FFA	positive	Pinolenic acid	C18H30O2	279.2324	278.2246	8.5318	M + H	0.9153	2	0.7256	up	down	0.001
		positive	Isopalmitic acid	C16H32O2	279.2326	256.2402	9.3213	M + Na	6.2597	2	0.5484	up	down	0.002
		negative	Tetradecanedioic acid	C14H26O4	257.1757	258.1831	7.1493	M-H	2.5835	1	0.9466	up	down	0.000
		negative	16-Hydroxyhexadecanoic acid	C16H32O3	271.2278	272.2351	8.5644	M-H	1.3513	1	0.9254	up	down	0.000
		negative	Epoxyoleic acid	C18H34O3	297.2435	298.2508	9.0433	M-H	2.7730	1	0.9601	up	down	0.001
	Others	positive	7-[3-(Undeca-2,5-dien-1-yl)oxiran-2-yl]hept-5-enoic acid	C20H32O3	343.223	320.2351	9.154	M + Na	2.3247	2	0.8686	up	down	0.001
		positive	Palmitoleoyl Ethanolamide	C18H35NO2	298.2745	297.2668	6.3697	M + H	3.5328	2	0.8858	up	down	0.001
	Oxidized lipids	negative	(±)8-HETE	C20H32O3	379.2463	320.2351	9.0572	M + CH3COO	8.1341	1	0.7358	up	down	0.001
		negative	17-HDoHE	C22H32O3	343.2276	344.2351	8.7657	M-H	0.7185	1	0.7538	up	down	0.001
		negative	14,15-Epoxy-5,8,11-eicosatrienoic acid	C20H32O3	319.2273	320.2351	9.8658	M-H	1.3750	3	0.8233	up	down	0.017
		negative	5,6-EET	C20H32O3	319.2271	320.2351	10.2498	M-H	1.8750	3	0.5956	up	down	0.023
		negative	13-Hpode	C18H32O4	311.2225	312.2301	8.6596	M-H	1.4136	1	0.8843	up	down	0.003
		negative	13R-hydroxy-9Z,11E-octadecadienoic acid	C18H32O3	295.2276	296.2351	8.4673	M-H	0.0292	1	0.9092	up	down	0.000
		negative	13-HOTE	C18H30O3	353.2306	294.2195	8.5526	M + CH3COO	7.1813	1	0.7807	up	down	0.000
GL	MG	positive	MG(0:0/18:2(9Z,12Z)/0:0)	C21H38O4	377.2666	354.277	10.6536	M + Na	2.3948	2	0.9455	down	up	0.049
	DG	positive	1-Arachidonoyl-3-docosahexaenoyl-sn-glycerol	C45H68O5	689.5241	688.5067	10.124	M + H	13.5161	3	0.5664	up	down	0.022
GP	LPA	negative	LysoPA(24:1(15Z)/0:0)	C27H53O7P	501.3396	520.3529	8.2938	M-H2O-H	8.7608	3	0.6181	down	up	0.005
	LPC	positive	LPC(16:1/0:0)	C24H48NO7P	494.3244	493.3168	7.9857	M+	1.9056	1	0.8125	down	up	0.002
	LPE	positive	LPE(16:1/0:0)	C21H42NO7P	452.2773	451.2699	7.9401	M + H	0.6677	1	0.8748	down	up	0.003
		negative	Glycerophospho-N-Arachidonoyl Ethanolamine	C25H44NO7P	500.2801	501.2855	8.2482	M-H	6.1317	1	0.8564	up	down	0.016
		negative	LPE(0:0/20:3)	C25H46NO7P	502.2934	503.3012	8.6139	M-H	13.3091	1	0.8117	up	down	0.006
		negative	LPE(0:0/22:6)	C27H44NO7P	524.2795	525.2855	8.2179	M-H	3.8829	1	0.7304	up	down	0.038
	LPS	negative	LPS(18:3/0:0)	C24H42NO9P	578.2819	519.2597	9.6926	M + CH3COO	13.4776	1	0.641	down	up	0.000
	PC	positive	LPC(15:0/0:0)	C23H48NO7P	482.3242	481.3168	8.1836	M + H	1.5363	1	0.8707	down	up	0.009
		positive	[2-hydroxy-3-[(9Z,12Z)-octadeca-9,12-dienoyl]oxypropyl] 2-(trimethylazaniumyl)ethyl phosphate	C26H50NO7P	542.3223	519.3325	8.3652	M + Na	0.4088	2	0.9414	down	up	0.000
		positive	1-octadecanoyl-2-(4Z,7Z,10Z,13Z,16Z,19Z-docosahexaenoyl)-sn-glycero-3-phosphocholine	C48H84NO8P	834.6151	833.5935	10.6082	M + H	17.7987	2	0.6782	up	down	0.023
		positive	1-Myristoleoyl-2-(1-enyl-palmitoyl)-sn-glycero-3-phosphocholine	C38H74NO7P	688.5211	687.5203	10.124	M + H	11.4470	3	0.5594	up	down	0.007
		negative	PC(16:0/22:4(7Z,10Z,13Z,16Z))	C46H84NO8P	790.5608	809.5935	8.6454	M-H2O-H	18.6699	3	0.5133	up	down	0.002
		negative	1-Palmitoyl-2-(1-enyl-stearoyl)-sn-glycero-3-phosphocholine	C42H84NO7P	766.5629	745.5985	8.6139	M + Na-2H	9.7064	3	0.5431	up	down	0.000
	PE	negative	PE-NMe2(18:1(11Z)/18:1(11Z))	C43H82NO8P	806.5569	771.5778	8.6596	M + Cl	12.0142	3	0.5633	up	down	0.003
		positive	1-(9Z-heptadecenoyl)-sn-glycero-3-phosphoethanolamine	C22H44NO7P	466.2933	465.2855	8.4559	M + H	1.9958	2	0.8677	down	up	0.014
Heterocyclic compounds	Heterocyclic compounds	positive	6-Chloropurine	C5H3ClN4	137.002	154.0046	1.1033	M + H-H2O	1.4811	2	0.7566	down	up	0.041
		positive	Janthitrem B	C37H47NO5	568.3379	585.3454	8.6076	M + H-H2O	8.6088	3	0.701	up	down	0.030
		negative	5-(3,4-Diacetoxybut-1-ynyl)-2,2′-bithiophene	C16H14O4S2	370.9908	334.0334	10.1268	M + K-2H	21.6136	3	0.7508	down	up	0.002
		positive	Silodosin	C25H32F3N3O4	496.241	495.2345	7.9401	M + H	2.4414	2	0.8902	down	up	0.011
		positive	Bispyribac	C19H18N4O8	499.1043	430.1125	4.4882	M + Na + HCOOH	5.5239	2	0.6979	up	down	0.010
		positive	Retusin	C19H18O7	359.1042	358.1053	2.4532	M + H	20.6135	2	0.6569	down	up	0.000
		positive	Dexanabinol	C25H38O3	387.2899	386.2821	8.6989	M + H	1.6670	2	0.9174	up	down	0.009
		positive	tamoxifen N-beta-D-glucosiduronic acid	C32H38NO7+	548.2738	548.2648	8.547	M+	12.5058	3	0.5604	up	down	0.008
		negative	Bropirimine	C10H8BrN3O	323.9963	264.9851	1.74	M + CH3COO	5.1490	2	0.7372	up	down	0.040
		negative	Hesperidin	C28H34O15	626.2199	610.1898	8.7512	M + NH4-2H	19.7797	2	0.6419	up	down	0.034
		negative	3,6,8-Trimethylallantoin	C7H12N4O3	252.0876	200.0909	2.7688	M + Cl + NH3	1.7750	3	0.7307	up	down	0.026
	Indole and Its derivatives	positive	5-Methoxyindoleacetate	C11H11NO3	206.0815	205.0739	4.1352	M + H	0.3860	1	0.9154	down	up	0.004
		negative	benzyl 4-(1,3-dioxoisoindolin-2-yl)-2′,2′-dimethyl-3′,5-dioxo-2′,3′,4,5-tetrahydro-3H-spiro[furan-2,9′-imidazo[1,2-a]indole]-1’(9a’H)-carboxylate	C31H25N3O7	550.162	551.1693	4.1964	M-H	0.4938	2	0.8413	down	up	0.011
Hormones and hormone related compounds	Hormones and hormone related compounds	positive	[4-[(4-acetamidobenzoyl)amino]phenyl] (Z)-7-[(1R,2R,3R)-3-hydroxy-2-[(E,3R)-3-hydroxy-4,4-dimethyloct-1-enyl]-5-oxocyclopentyl]hept-5-enoate	C37H48N2O7	615.3439	632.3462	9.3062	M + H-H2O	0.3513	2	0.7039	down	up	0.045
		negative	Ergosta-5,7-dien-3beta-ol	C28H46O	433.3316	398.3549	9.7847	M + Cl	16.9157	3	0.6925	up	down	0.001
		negative	Prostaglandin h2	C20H32O5	351.2157	352.225	9.3966	M-H	4.9250	3	0.6104	up	down	0.009
		negative	Zymosterol	C27H44O	436.3383	384.3392	8.2938	M + Cl + NH3	7.1734	3	0.8675	down	up	0.019
		negative	Cytochalasin Opho	C28H37NO4	450.2624	451.2723	7.6507	M-H	6.1481	3	0.6168	down	up	0.028
		negative	(10S)-Juvenile hormone III diol	C16H28O4	305.1734	284.1988	7.6814	M + Na-2H	0.1500	3	0.9321	up	down	0.003
Nucleotide and Its metabolites	Nucleotide and Its metabolites	positive	Flavin mononucleotide	C17H21N4O9P	457.1121	456.1046	3.2347	M + H	0.3938	3	0.581	down	up	0.011
		negative	Cytidine-5′-diphosphate	C9H15N3O11P2	384.0001	403.0182	4.454	M-H2O-H	0.1497	2	0.8758	up	down	0.006
		negative	2-Deoxyribose-5′-phosphate	C5H11O7P	273.038	214.0242	0.8091	M + CH3COO	0.6472	1	0.8813	up	down	0.001
		negative	(L-alanyl)adenylate	C13H19N6O8P	458.1097	418.1002	1.1601	M-H + CH3CN	20.1258	3	0.6848	up	down	0.015
Organic acid and Its derivatives	Organic acid and Its derivatives	positive	Pipecolic acid	C6H11NO2	130.0861	129.079	0.6209	M + H	0.1133	1	0.9333	up	down	0.036
		positive	Lenticin	C14H18N2O2	291.0981	246.1368	5.2079	M-H + 2Na	22.1812	2	0.7788	up	down	0.006
		positive	Indolepyruvate	C11H9NO3	204.0655	203.0582	4.1352	M + H	0.1000	3	0.9545	down	up	0.000
		positive	N-(indole-3-acetyl)-L-leucine	C16H20N2O3	333.1236	288.1474	4.1352	M-H + 2Na	12.2750	3	0.7719	down	up	0.001
		positive	5,6-DHET	C20H34O4	339.2516	338.2457	8.3802	M + H	4.3247	1	0.6942	up	down	0.003
		positive	6-({2-[4,6-dihydroxy-2-methoxy-3-(3-methylbut-2-en-1-yl)phenyl]-2-hydroxyacetyl}oxy)-3,4,5-trihydroxyoxane-2-carboxylic acid	C20H26O12	459.1526	458.1424	5.2079	M + H	9.9477	3	0.5259	up	down	0.002
		negative	2-Hydroxymyristic acid	C14H28O3	243.1963	244.2038	7.4375	M-H	0.7212	2	0.936	up	down	0.000
		negative	3-Hydroxydodecanoic acid	C12H24O3	215.1649	216.1725	7.7579	M-H	0.8500	3	0.851	up	down	0.008
		negative	S-nitroso-L-cysteinylglycine	C5H9N3O4S	247.0482	207.0314	0.8248	M-H + CH3CN	4.7500	3	0.785	up	down	0.011
		negative	Azelaic acid	C9H16O4	187.0973	188.1049	4.5294	M-H	0.6500	3	0.8129	down	up	0.003
		negative	FFA(9:0)	C9H18O2	315.2539	158.1307	8.3089	2 M-H	0.7406	1	0.7784	up	down	0.003
		negative	2-Propyl-2-pentenoic acid	C8H14O2	283.1914	142.0994	7.6879	2 M-H	0.5628	1	0.764	up	down	0.004
		negative	19-Hydroxyarachidonic acid	C20H32O3	319.2276	320.2351	8.5066	M-H	0.6250	3	0.8256	up	down	0.000
		negative	2-((4-Pyridinylcarbonyl)hydrazono)pentanedioic acid	C11H11N3O5	305.0817	265.0699	6.4023	M-H + CH3CN	17.2250	3	0.5927	down	up	0.013
		negative	1-Oleoylglycerone 3-phosphate	C21H39O7P	450.2627	434.2433	7.8653	M + NH4-2H	3.6616	3	0.7057	down	up	0.001
	Phosphoric acids	positive	Heptenophos	C9H12ClO4P	251.0135	250.0162	5.6979	M + H	24.3453	2	0.6359	up	down	0.006
		positive	Phosphocholine	C5H15NO4P+	184.0736	184.0739	0.7439	M + H	0.9637	1	0.8645	down	up	0.000
		negative	Copal-8-ol diphosphate	C20H38O8P2	508.2212	468.2042	7.8653	M-H + CH3CN	3.3647	3	0.6413	down	up	0.000
	Sulfonic acids	negative	Lithocholyltaurine	C26H45NO5S	482.2953	483.3018	9.531	M-H	0.8897	3	0.7555	up	down	0.013
SL	Cer	positive	CerP(d18:1/12:0)	C30H60NO6P	544.406	561.4158	7.8789	M + H-H2O	14.5826	2	0.6477	up	down	0.002
Terpenoids	Ditepenoids	negative	Phorbol caprate, tiglate	C35H52O8	599.369	600.3662	10.3573	M-H	14.9741	3	0.5373	down	up	0.021
Tryptamines, Cholines, Pigments	Tryptamines	negative	(9Z)-N-[2-(5-hydroxy-1H-indol-3-yl)ethyl]octadec-9-enamide	C28H44N2O2	439.3369	440.3403	9.6617	M-H	9.2607	2	0.7062	down	up	0.001
Others	Medicine	positive	Trazodone hydrochloride	C19H23Cl2N5O	407.1237	407.128	4.1352	M+	7.3252	1	0.6408	down	up	0.005
	Others	negative	[(2R,3S,4R,5R)-5-(4-amino-2-oxopyrimidin-1-yl)-3,4-dihydroxyoxolan-2-yl]methyl [hydroxy-[2-(trimethylazaniumyl)ethoxy]phosphoryl] phosphate	C14H26N4O11P2	504.1359	488.1073	1.9219	M + NH4-2H	18.3882	3	0.7775	down	up	0.000
		negative	Chymostatin	C31H41N7O6	606.3112	607.3118	9.1873	M-H	7.2288	2	0.6198	down	up	0.001
		negative	6-({1-[(2R)-3-[2-amino-3-methyl-4-(2-methyl-1,3-thiazol-4-yl)but-3-en-1-yl]-2-methyloxiran-2-yl]-10-carboxy-9-hydroxy-4,6,8,8-tetramethyl-7-oxodecan-5-yl}oxy)-3,4,5-trihydroxyoxane-2-carboxylic acid	C33H52N2O12S	716.3387	700.3241	7.9113	M + NH4-2H	8.1581	3	0.537	down	up	0.038

*p* represents the difference between the model group and the XZK group.

### 3.4 Correlation of rescued metabolites with inflammation and lipid serum levels

**Figure 4:**
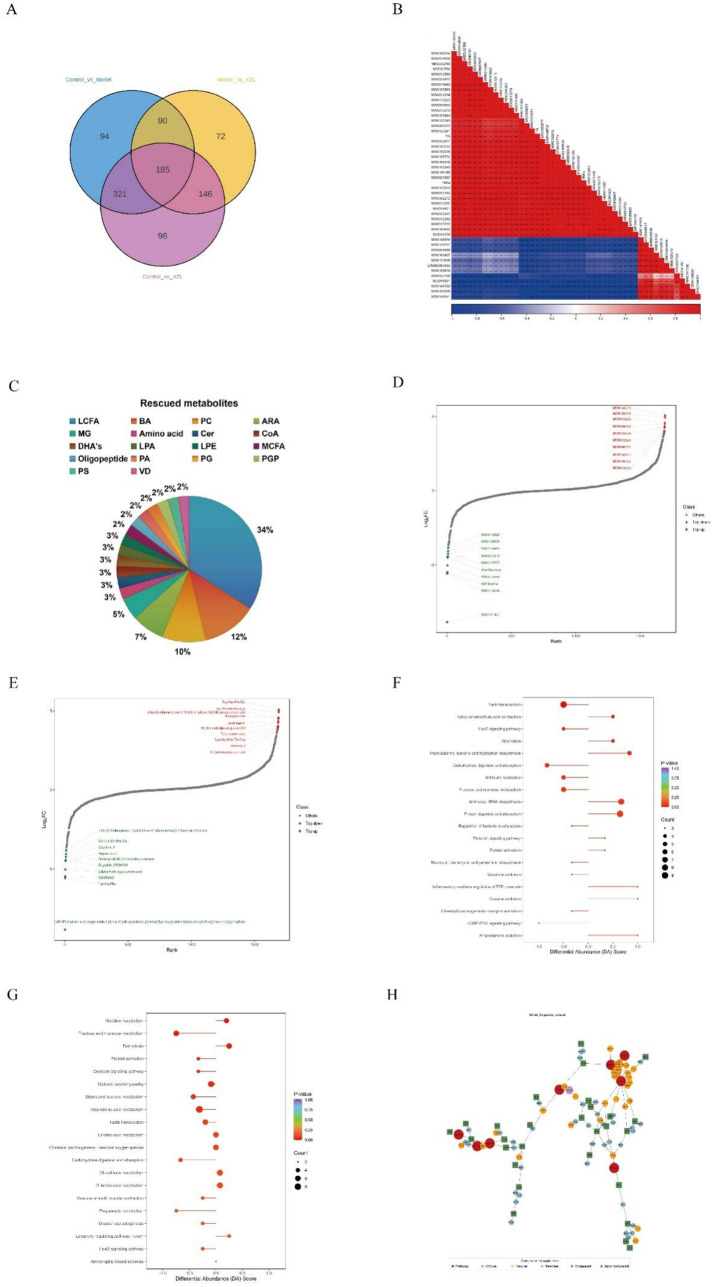
Summary of differential metabolite classification and related pathway analysis. **(A)** The Venn diagram among three groups. **(B)** Heatmap illustrating the correlation between TNF-*α*, TG in serum and the metabolites rescued by XZK in liver samples from the CON, MOD, and XZK groups. **(C)** Classification of interesting metabolites. **(D)** The FC distribution of significantly changed metabolites between CON vs. MOD. **(E)** The FC distribution of significantly changed metabolites between XZK vs. MOD. **(F)** KEGG pathway enrichment analysis of the different metabolites between MOD and CON. **(G)** KEGG pathway enrichment analysis of the different metabolites between MOD and XZK. **(H)** Regulation network of different metabolites.

Common metabolites with differential expressions through pairwise comparison was shown in Figure 4A. We explored the correlation between the metabolites rescued by XZK and the levels of TNF-*α* and TG, both of which are critical markers of inflammation and lipid accumulation in NAFLD. The correlation coefficients between TNF-*α*, TG, and the metabolites rescued by XZK reveal a strong correlation between many of the rescued metabolites and these two key pathological markers (Figure 4B), suggesting that these metabolites may mediate the dual therapeutic effects of XZK on inflammation and lipid metabolism.

Analysis of these metabolites revealed distinct classes associated with each pathological process (Figure 4C). On one hand, 14 long-chain fatty acids (LCFAs) showed elevated levels in the MOD group, indicative of hepatic lipid accumulation, and were significantly reduced by XZK treatment. This finding is consistent with the oil red O staining results (Figures 1A,B), which showed reduced lipid deposition in XZK-treated livers. On the other hand, 3 metabolites associated with arachidonic acid metabolism, three arachidonic acid-related metabolites, which are key precursors of pro-inflammatory mediators, were elevated in the MOD group and significantly reduced following XZK treatment (Figures 4D,E). This reduction paralleled the decrease in serum TNF-*α* levels (Supplementary Figures 3A,B). Additionally, 5 metabolites related to bile acid metabolism were restored by XZK, suggesting improved hepatic excretory function.

The strong correlation between these metabolite classes and their respective pathological markers further supports this interpretation: LCFAs correlated closely with TG levels, while arachidonic acid metabolites correlated with TNF-*α*. These findings indicate that XZK simultaneously targets both lipid accumulation and inflammation through modulation of distinct metabolic pathways. This dual action provides a metabolic basis for the coordinated improvement in hepatic steatosis and inflammatory status observed in XZK-treated animals.

### 3.5 Potential signaling pathway regulated by XZK

To identify the systemic metabolic pathways perturbed in NAFLD and targeted by XZK, we performed KEGG pathway enrichment analysis. This analysis revealed that XZK primarily modulates pathways central to core energy metabolism, including carbon metabolism, 2-oxocarboxylic acid metabolism, and biosynthesis of amino acids and nucleotides (Figures 4F–H). In NAFLD, dysfunction in these hubs—often driven by nutrient overload—leads to mitochondrial stress, oxidative burden, and accumulation of bioactive lipids. Notably, these metabolic stresses are well-established upstream activators of stress-responsive kinases, including JNK. Therefore, the significant enrichment of these pathways provides a plausible metabolic context for the observed suppression of the JNK/AP-1/TNF-*α* axis by XZK, suggesting that its anti-inflammatory effects may be secondary to the restoration of hepatic metabolic homeostasis.

We focused on carbon metabolism as a central hub, given its fundamental role in bioenergetics and biosynthetic precursor supply. Notably, dysregulated hepatic carbon metabolism is a hallmark of metabolic syndrome and NAFLD, often driven by excessive dietary sugar (e.g., fructose), which promotes *de novo* lipogenesis and oxidative stress. Importantly, disturbances in carbon metabolism are known upstream activators of stress-responsive signaling pathways. For instance, prior studies have shown that metabolic stress can induce cytochrome P450 enzymes and activate genes such as *cFos* and *cJUN*, leading to increased activity of the nuclear transcription factor AP-1 (22). This is highly relevant, as elevated AP-1 DNA-binding activity has been consistently documented in patients with NASH and is recognized as a key regulator in NAFLD pathogenesis (23).

Integrating our metabolomic findings with this established knowledge, we propose a coherent mechanistic model: XZK, by rectifying the dysregulated carbon metabolism as evidenced by the normalization of key metabolites in this pathway, likely reduces the underlying metabolic stress that chronically activates stress kinases like JNK. This, in turn, would lead to decreased phosphorylation and activation of AP-1 components (cJUN/cFos), ultimately suppressing the transcription of pro-inflammatory and fibrogenic target genes, such as TNF-*α*. This model positions the AP-1 signaling pathway as a critical transcriptional effector downstream of the metabolic improvements orchestrated by XZK, connecting our biochemical observations to a well-defined inflammatory regulatory axis in NAFLD. Furthermore, considering the role of the gut-liver axis in modulating both metabolic and inflammatory signals (24), future studies should investigate whether XZK’s impact on carbon metabolism and AP-1 involves gut-derived mediators.

### 3.6 XZK regulated JNK/AP-1/TNF-*α* signaling pathway

To investigate the involvement of the JNK/AP-1/TNF-*α* signaling pathway in the therapeutic effects of XZK on NAFLD, we assessed the mRNA expression and immunohistochemical (IHC) staining of key pathway components, including cFos, cJUN, JAK1/2/3, and TNF-*α*, in liver tissues from all experimental groups.

**Figure 5:**
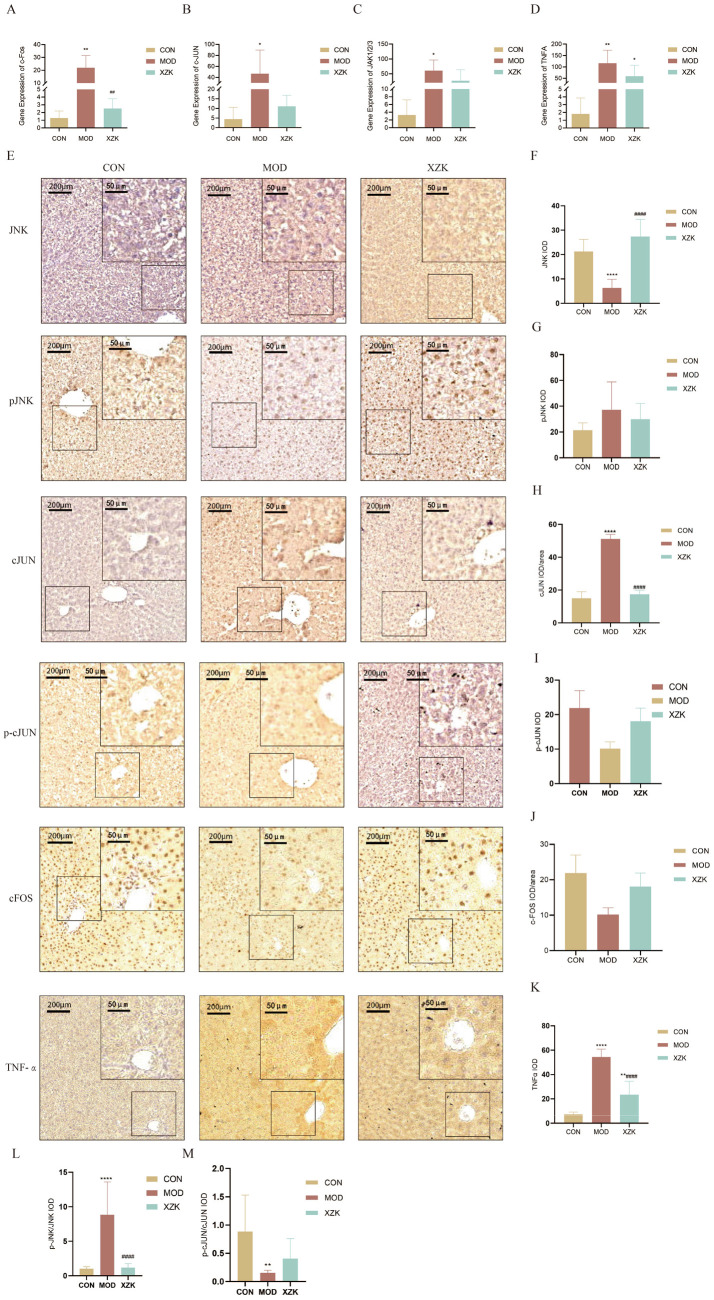
Gene expression and immunohistochemistry of core metabolites in liver. **(A)** Gene expression of cFos in liver. **(B)** Gene expression of cJUN in liver. **(C)** Gene expression of JAK1/2/3 in liver. **(D)** Gene expression of TNF-*Α* in liver. **(E)** Representative IHC staining images of CON, MOD, and XZK group (bar = 200 μm and 50 μm). **(F–K)** Statistical analysis of JNK, p-JNK, cJUN, p-cJUN, cFOS, TNF-*α* in the liver tissues (*n* = 9). **(L)** Quantification of p-JNK/total JNK ratio (*n* = 9). **(M)** Quantification of p-JUN/total JUN ratio (*n* = 9). *p < 0.05, **p < 0.01, ***p < 0.001, ****p < 0.0001 via CON group; #*p* < 0.05, ##*p* < 0.01 via the MOD group.

Gene expression analyses revealed that the JNK/AP-1/TNF-*α* signaling pathway is activated in NAFLD and modulated by XZK treatment. As shown in Figures 5A–C, HFD feeding significantly upregulated the mRNA levels of cFos (*p* < 0.01), cJUN, and JAK1/2/3 compared to the CON group, indicating pathway activation. XZK treatment significantly attenuated these elevations, reducing the expression of cFos, cJUN, and JAK1/2/3 toward normal levels. Similarly, TNF-*α* mRNA expression, a downstream target of AP-1 transcriptional activity, was markedly increased in the MOD group and significantly reduced by XZK treatment (*p* < 0.05; Figure 5D).

Immunohistochemical analysis provided further insights into pathway activation at the protein level. As shown in Figures 5E–M, XZK treatment decreased the immunoreactivity of cFos, cJUN, and TNF-*α* in liver sections compared to the MOD group. To assess pathway activation status, we quantified the ratio of phosphorylated to total protein for JNK and cJUN. The results revealed that the p-JNK/total JNK ratio was significantly elevated in the MOD group and markedly reduced by XZK treatment (*p* < 0.01), indicating suppression of upstream kinase activation (Figure 5L). In contrast, the p-cJUN/total cJUN ratio showed a distinct pattern: it was significantly decreased in the MOD group compared to CON (*p* < 0.01), and was elevated by XZK treatment, returning toward normal levels (Figure 5M). This differential regulation suggests a complex modulation of AP-1 subunit phosphorylation, potentially reflecting distinct roles of cJUN *versus* other AP-1 components in the context of XZK treatment. Nevertheless, the consistent reduction in TNF-*α* expression—at both mRNA and protein levels—indicates that the net effect of XZK on AP-1 transcriptional output is inhibitory.

## 4 Discussion

In this study, we developed a NAFLD model by administering a HFD to hamsters for 8 weeks. The lipoprotein metabolism in golden hamsters closely resembles that of humans, including the expression of plasma cholesterol ester transfer protein (CETP), which can lead to a significant increase in total cholesterol/triglyceride (CHO/TG) levels. During the advanced stages of induction, symptoms associated with NASH manifest, accompanied by histopathological features such as ballooning degeneration, inflammatory cell infiltration, and fibrosis, which is essential for our research on XZK’s effects on NAFLD. Our findings demonstrated that treatment with RYRE (XZK) significantly reduced serum levels of TC, TG, and LDL-C, while increasing HDL-C levels. RYRE also effectively mitigated hepatic lipid accumulation. Notably, no significant pathological damage such as severe ballooning or bridging fibrosis was observed in the liver tissue of HFD-fed hamsters, which may be attributed to the relatively short induction period, resulting in lipid accumulation without advanced hepatocyte injury. Furthermore, XZK modulated serum levels of AST, ALB, TBiL, and DBiL. Simvastatin (SVT), used as a positive control, inhibits cholesterol synthesis in T cells (25), regulates redox enzymes (26), activates anti-inflammatory pathways (27), and reduces liver macrophage numbers (28), which aligns with our observed effects of SVT in reducing serum TG, modulating inflammatory factors (IL-4, IL-1*β*, TGF-*β*), and improving hepatic lipid deposition.

To delve deeper into the mechanism, we performed untargeted metabolomics analysis coupled with KEGG pathway enrichment analysis. A total of 135 differential metabolites were identified between the MOD and XZK groups. Pathway enrichment revealed that these metabolites were most significantly clustered in core metabolic hubs, including carbon metabolism, 2-oxocarboxylic acid metabolism, amino acid biosynthesis, and nucleotide metabolism (Figure 4G). This global metabolic reprogramming suggests that XZK treatment rectifies the fundamental bioenergetic and biosynthetic disturbances in NAFLD. Critically, several of these altered metabolic pathways are known upstream regulators of stress-responsive signaling cascades. For instance, disturbances in carbon and lipid metabolism can lead to the accumulation of bioactive metabolites and cellular stress—potent activators of the JNK pathway. Given that JNK is a major upstream kinase that phosphorylates and activates AP-1 components (cJUN), our metabolomic findings provide a plausible metabolic trigger for the observed modulation of the AP-1 pathway. Thus, the metabolomic profile under XZK treatment not only reflects improved metabolic homeostasis but also points to a reduction in the metabolic drivers that activate pro-inflammatory pathways like JNK/AP-1.

The normalization of these core metabolic pathways provides a plausible upstream context for the observed suppression of the JNK/AP-1/TNF-*α* axis. Disruptions in carbon and lipid metabolism, particularly those induced by high-fat and high-fructose diets, are known to generate cellular stress signals, including oxidative stress and endoplasmic reticulum (ER) stress, both of which are potent activators of stress-responsive kinases such as JNK (22, 29). For instance, fructose metabolism overloads hepatic mitochondria and promotes *de novo* lipogenesis, leading to the accumulation of reactive oxygen species and bioactive lipids that directly trigger JNK phosphorylation (29). In the present study, XZK treatment significantly restored metabolites in carbon metabolism and normalized multiple lipid species, including long-chain fatty acids and arachidonic acid-derived mediators. These metabolic improvements likely alleviate the upstream stress signals that drive JNK activation, thereby attenuating downstream AP-1 transcriptional activity and TNF-*α* production. Thus, rather than viewing the metabolomic changes as independent from the inflammatory phenotype, our findings position them as metabolic triggers whose correction by XZK leads to consequent suppression of pro-inflammatory signaling.

**Figure 6:**
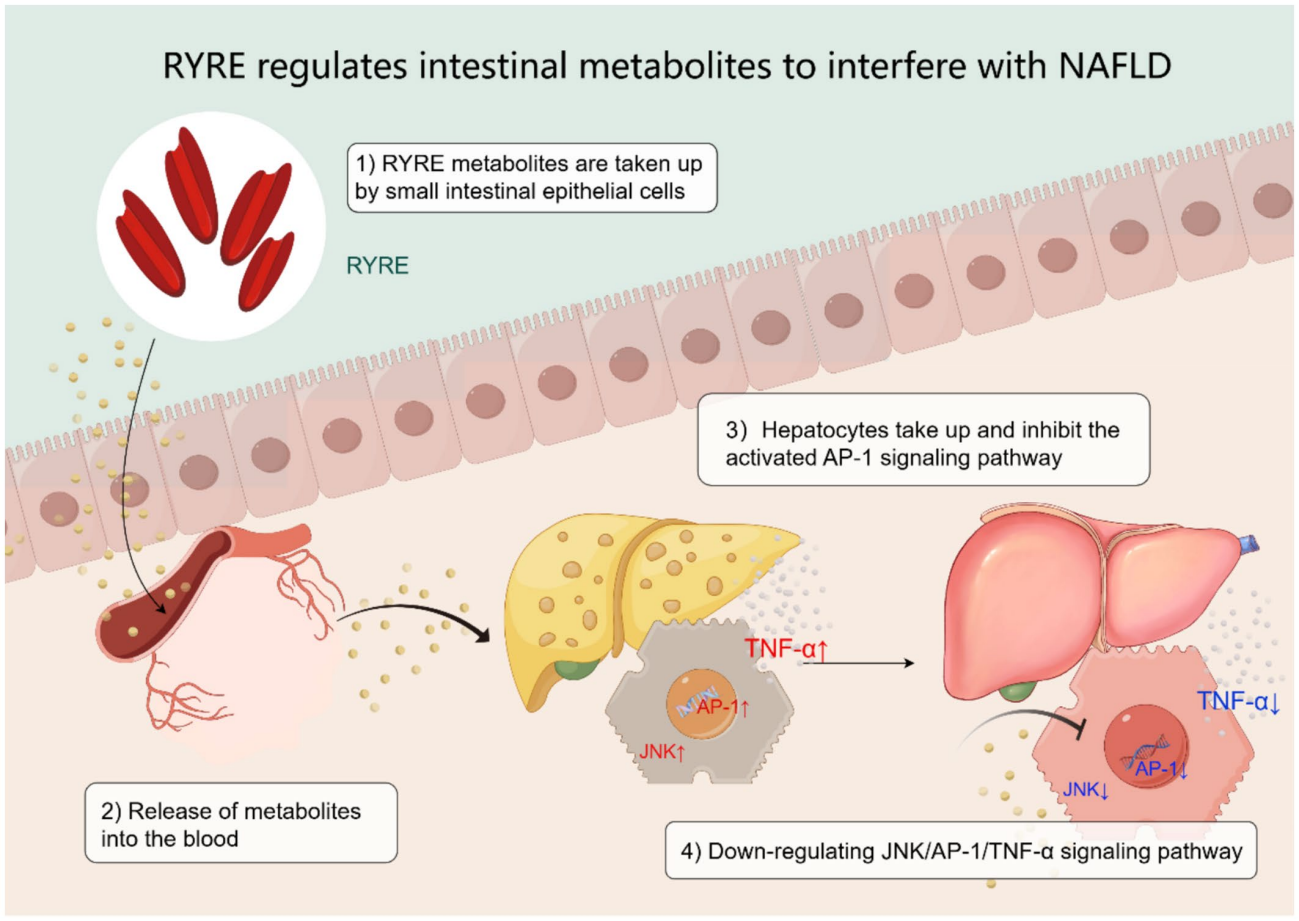
Possible mechanisms of action of RYRE on NAFLD.

Building upon this metabolic context, our subsequent investigations focused on the AP-1 transcriptional complex, a pivotal regulator of inflammation and cell fate. We observed that XZK significantly influenced AP-1 dynamics in the liver. Our data suggest a functional antagonism between different AP-1 dimers. Specifically, while AP-1 activity is central to the disease process, XZK appears to modulate its composition (Figure 6). cJUN/cFos dimers were increased, whereas other cJUN-containing dimers associated with TNF-*α* expression were reduced. This suggests a partner-dependent effect of cJUN on the TNF-*α* promoter. Previous studies in other organs suggested overlapping functions of different Fos proteins (30, 31). We here identify the TNF-*α* promoter as being antagonistically regulated by different AP-1 dimer compositions. This raises an intriguing question regarding how structurally similar complexes exert opposite effects, which may involve differential recruitment of co-regulators such as Sirt1 or HDAC3, known players in hepatic lipid metabolism (32). The correlation between AP-1 component modulation and attenuation of the JNK/AP-1/TNF-*α* signaling axis underscores AP-1’s role as a significant regulatory node in NAFLD. Since HFD activates multiple upstream pathways like insulin, JNK, PKC pathways (33), which converge on AP-1, our findings position AP-1 as a key integrator of metabolic and inflammatory stress, which is effectively targeted by XZK. Our findings, which demonstrate that XZK ameliorates hepatic steatosis, inflammation, and metabolic dysregulation via the AP-1 pathway in a hamster model of NAFLD, provide a mechanistic substrate for its documented clinical benefits. The observed modulation of key serum lipids and inflammatory markers in our animal study aligns with the outcomes reported in clinical trials of XZK for hyperlipidemia and NAFLD (17, 34). Thus, the mechanistic insights gained here strengthen the biological plausibility of its use in human metabolic disease and highlight potential biomarkers for future clinical validation.

Despite the insights gained, this study has several limitations that should be considered. First, the sample size, though adequate for an exploratory metabolomics and pharmacological study, was relatively small (*n* = 10 per group), which may affect the statistical robustness of some endpoints. Additionally, the HFD-induced hamster NAFLD model, while validated, does not encompass the full etiological spectrum of human NAFLD, which involves genetic, gut microbiota, and lifestyle factors. Future studies with larger cohorts are warranted to confirm our findings. Employing other preclinical models (e.g., methionine-choline deficient diet models, genetic models) or moving toward clinical biomarker validation in patient cohorts would enhance the translational relevance of our results. Secondly, our investigation of the JNK/AP-1/TNF-*α* signaling pathway relied on mRNA measurement and immunohistochemistry. More direct quantification of key protein expressions and phosphorylation states via techniques like Western blot would provide stronger mechanistic evidence. Moreover, the absence of *in vitro* experiments limits our ability to attribute the observed effects to direct actions of RYRE on hepatocytes or immune cells versus secondary systemic effects. Subsequent research should include comprehensive protein-level analyses and employ *in vitro* systems to directly interrogate the role of RYRE or its specific components in modulating lipid metabolism, inflammation, and the AP-1 pathway in a controlled cellular environment. Thirdly, while our untargeted metabolomics successfully identified numerous altered metabolites, the study design cannot establish causality between specific metabolic shifts and the therapeutic benefits of RYRE. Targeted metabolomics or isotopic flux analysis focused on the highlighted pathways is needed to validate and quantify these changes. Functional experiments modulating key identified metabolites or their associated enzymes could help establish causal links. Fourth, our study design lacked an XZK-only control group (XZK administration to healthy hamsters on a normal diet). This limits our ability to distinguish the direct, intrinsic hepatic effects of XZK from its therapeutic effects in the context of HFD-induced pathology and precludes a comprehensive assessment of its safety profile in a non-diseased state. Future studies aimed at evaluating the safety and potential metabolic effects of XZK in healthy organisms should include such a control group. Finally, RYRE (Xuezhikang) is a complex mixture. Our study treats it as a holistic intervention, and thus we cannot identify which specific compounds are primarily responsible for the observed effects. Fractionation of RYRE followed by bioactivity-guided isolation and testing of individual components would be a crucial next step to pinpoint the active principles, which is vital for drug development and quality control. Addressing these limitations in future work will deepen our understanding of RYRE’s therapeutic mechanisms and advance its potential application in NAFLD management. Nevertheless, the present study provides a solid foundation by integrating metabolomic profiling with *in vivo* validation, highlighting the AP-1 pathway as a promising target.

## 5 Conclusion

In this study, we employed a high-fat diet-induced hamster model of NAFLD to investigate the therapeutic mechanisms of the RYRE-based formulation, XZK. Integrating untargeted hepatic metabolomics with histopathological and biochemical analyses, we demonstrated that XZK effectively ameliorates hepatic steatosis, dyslipidemia, and inflammation.

Mechanistically, our findings reveal a multi-level therapeutic axis: XZK induces comprehensive reprogramming of hepatic metabolism—particularly rectifying disruptions in carbon metabolism, lipid metabolism, and bile acid homeostasis. This metabolic restoration is functionally linked to attenuation of the JNK/AP-1/TNF-*α* pro-inflammatory signaling pathway, as evidenced by qPCR and immunohistochemical analyses, including quantification of phosphorylated JNK. These results suggest that the hepatoprotective effects of XZK arise from concomitant restoration of metabolic homeostasis and suppression of AP-1-driven inflammation, with these processes being intrinsically interconnected.

In summary, this work provides a metabolomics-informed mechanistic perspective on XZK in NAFLD, moving beyond phenotypic observation to identify AP-1 as a pivotal transcriptional node. Future studies should focus on validating the causal role of specific metabolites in modulating the AP-1 pathway using *in vitro* models, conducting functional assays such as inhibitor studies to establish causality (e.g., SP600125), and translating these findings by investigating relevant metabolic and inflammatory biomarkers in clinical cohorts receiving XZK therapy.

## Data Availability

The data presented in the study are deposited in the China National Center For Bioinformation repository, accession number PRJCA061257.

## Footnotes

1
http://www.hmdb.ca/

2
http://www.genome.jp/kegg/

## Glossary

ALB: Albumin
ALP: Alkaline phosphatase
ALT: Alanine aminotransferase
ARA: Arachidonic acid metabolites
AST: Aspartate aminotransferase
BA: Bile acids and associated metabolites
BSA: Bovine serum albumin
CETP: Cholesterol ester transfer protein
CHO: Cholesterol
CHO/TG: total cholesterol/ triglyceride
CON: Control
DBiL: Direct bilirubin
ELISA: Enzyme-linked immunosorbent assay
HDL-C: High-density lipoprotein cholesterol
HE: Hematoxylin and eosin
MOD: Model
RYRE: Red yeast rice extract
HFCS: High-fructose corn syrup
HFD: High-fat diet
HMDB: Human Metabolome Database
IHC: Immunohistochemical
KEGG: Kyoto Encyclopedia of Genes and Genomes
LC/MS: Liquid chromatography-mass spectrometry
LCFA: Long-chain fatty acids
LDL-C: Low-density lipoprotein cholesterol
NAFLD: Non-alcoholic fatty liver disease
NASH: Non-alcoholic steatohepatitis
OPLS-DA: Orthogonal Partial Least Squares Discriminant Analyses
PBS: Phosphate buffered solution
PCA: Principal component analysis
QC: Quality control
qPCR: Quantitative polymerase chain reaction
SVT: Simvastatin
TBA: Total bile acid
TBiL: Total bilirubin
TCM: Traditional Chinese Medicine
TG: Triglycerides
T2DM: Type 2 diabetes mellitus
VIP: Variable Importance in Projection
XZK: Xuezhikang
ƴ-GT: Gamma-glutamyl transferase
