# Supplementary material for: Red yeast rice extract’s impact on liver health: a pharmacological and metabolomic exploration
Source: Front Nutr. 2026 Apr 15;13:1771594. doi: 10.3389/fnut.2026.1771594 (PMC13124985; doi:10.3389/fnut.2026.1771594)
Supplement: Supplementary file 1 [file Supplementary_file_1.docx]

**Supplementary Material**

Representative total ion chromatograms for the quality control (QC) samples showed in **Supplementary Figure 1.**

**
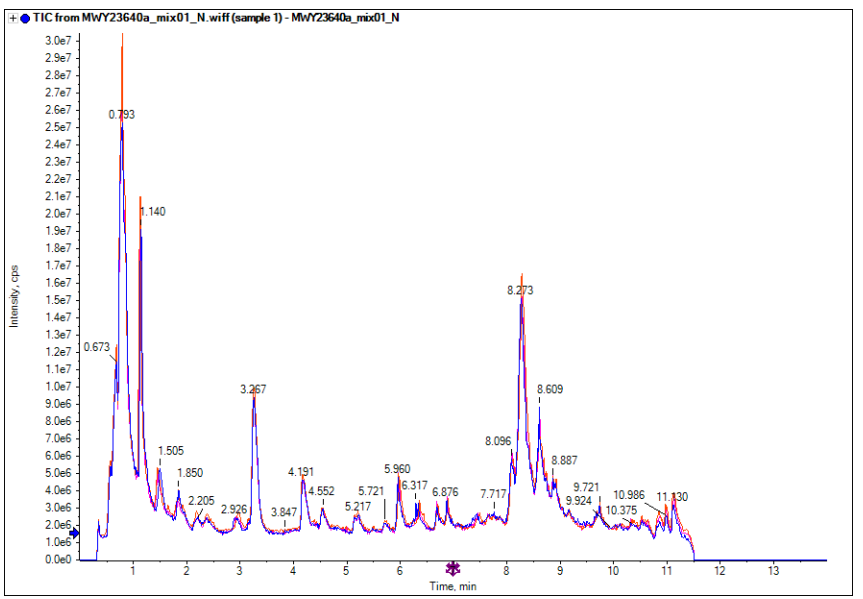
**

Orthogonal Partial Least Squares Discriminant Analyses (OPLS-DA) were conducted to identify potential biomarkers in **Supplementary Figure 2.**

**
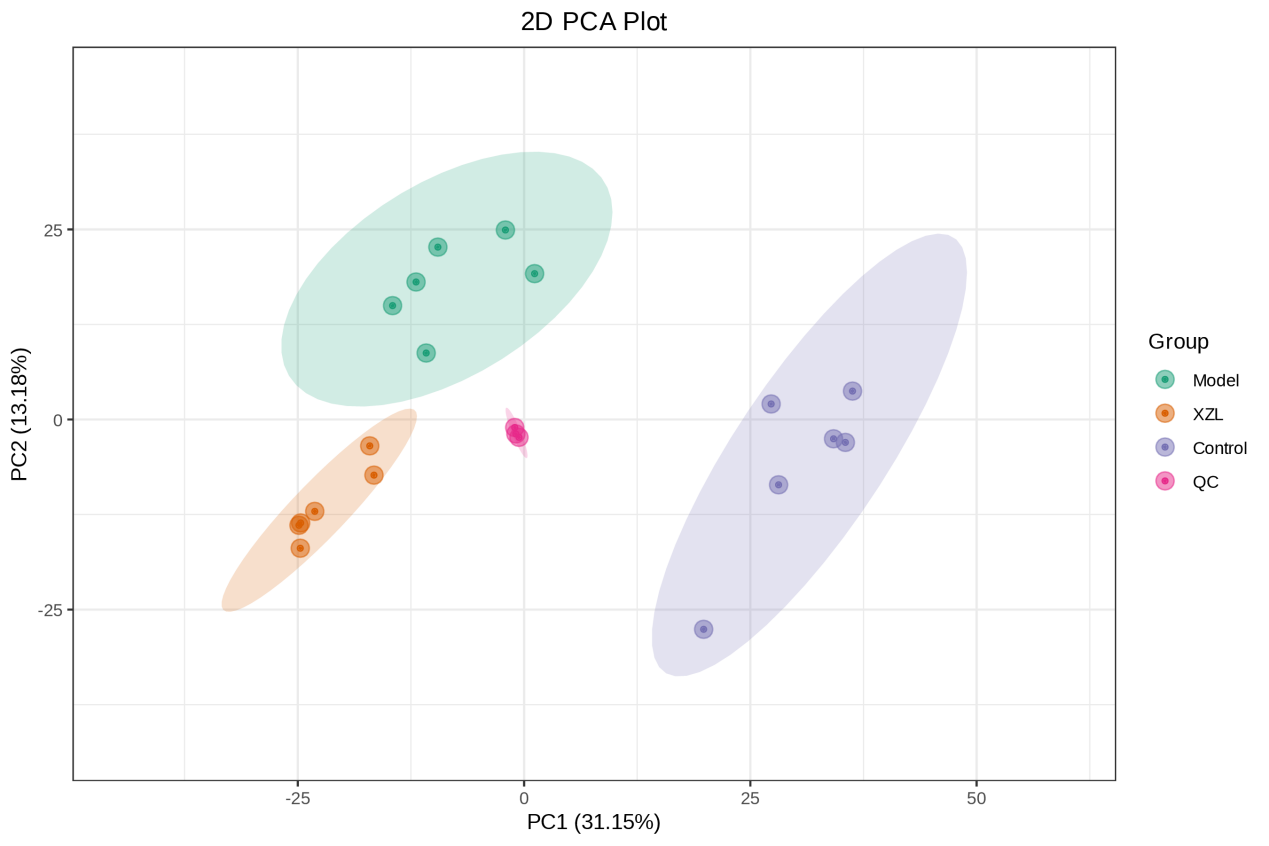
**

The changes in metabolites between MOD vs XZF were showed in **Supplementary Figure 3A-B.**

**
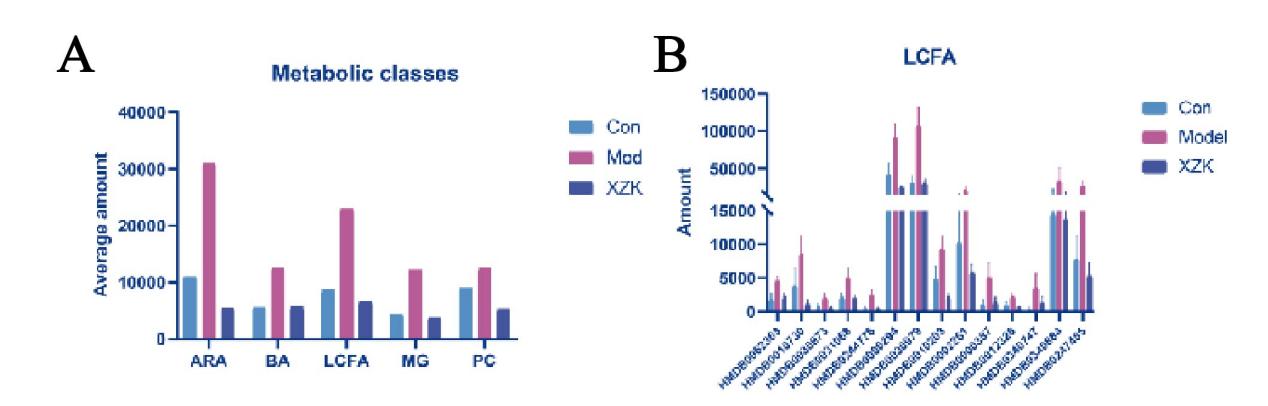
**

The metabolites detected in each group and the comparison among groups were showed in **Supplementary Table 1（Excel file）.**
